# First-step validation of a text message-based application for newborn clinical management among pediatricians

**DOI:** 10.1186/s12887-020-02307-2

**Published:** 2020-08-27

**Authors:** Santorino Data, Martin Mukama, Douglas McMillan, Nalini Singhal, Francis Bajunirwe

**Affiliations:** 1 Department of Pediatrics and Child Health, Mbarara University of Science and Technology, Mbarara, Uganda; 2 Consortium for Affordable Medical Technologies in Uganda, Mbarara, Uganda; 3 Department of Pediatrics, Dalhousie University, Halifax, Nova Scotia Canada; 4 Department of Pediatrics, University of Calgary, Calgary, Alberta Canada; 5 Department of Community Health, Mbarara University of Science and Technology, Mbarara, Uganda

**Keywords:** Newborn, mHealth, Phone application, Mortality, Morbidity, Birth attendant, Clinical management

## Abstract

**Background:**

Neonatal mortality is high in developing countries. Lack of adequate training and insufficient management skills for sick newborn care contribute to these deaths. We developed a phone application dubbed Protecting Infants Remotely by Short Message Service (PRISMS). The PRISMS application uses routine clinical assessments with algorithms to provide newborn clinical management suggestions. We measured the feasibility, acceptability and efficacy of PRISMS by comparing its clinical case management suggestions with those of experienced pediatricians as the gold standard.

**Methods:**

Twelve different newborn case scenarios developed by pediatrics residents, based on real cases they had seen, were managed by pediatricians and PRISMS®^.^ Each pediatrician was randomly assigned six of twelve cases. Pediatricians developed clinical case management plans for all assigned cases and then obtained PRISMS suggested clinical case managements. We calculated percent agreement and kappa (k) statistics to test the null hypothesis that pediatrician and PRISMS management plans were independent.

**Results:**

We found high level of agreement between pediatricians and PRISMS for components of newborn care including: 10% dextrose (Agreement = 73.8%), normal saline (Agreement = 73.8%), anticonvulsants (Agreement = 100%), blood transfusion (Agreement =81%), phototherapy (Agreement = 90.5%), and supplemental oxygen (agreement = 69.1%). However, we found poor agreement with potential investigations such as complete blood count, blood culture and lumbar puncture. PRISMS had a user satisfaction score of 3.8 out of 5 (range 1 = strongly disagree, 5 = strongly agree) and an average PRISMS user experience score of 4.1 out of 5 (range 1 = very bad, 5 = very good).

**Conclusion:**

Management plans for newborn care from PRISMS showed good agreement with management plans from experienced Pediatricians. We acknowledge that the level of agreement was low in some aspects of newborn care.

## Background

Over 90% of the global burden of neonatal mortality occurs in countries within resource limited settings [1]. Neonatal mortality accounted for about 40% of the under 5 mortality in 2015 [2]. Most neonatal deaths can be prevented by administration of proven interventions for newborn survival [3–6]. These interventions require the presence of skilled health workers to recognize a newborn in need of additional care, conduct a timely assessment, and establish an appropriate management plan [7].

Many health facilities in resource limited settings are understaffed and/or lack skilled manpower to provide appropriate health care including managing a sick newborn [8, 9].

In resource rich settings, neonatal mortality rate is low and neonatal care is a highly specialized discipline [7, 10]. Decisions regarding sick newborn care management in resource rich settings are most often made by highly qualified pediatricians or neonatologists [7]. However, in resource limited settings, the bulk of sick newborn care management decisions are made by frontline health workers (FLHW) including medical officers, nurses, and or midwives with no specialized neonatology training [9, 11, 12]. Some of these frontline cadres have not only inadequate training or experience to make management decisions for sick newborn care, but also have no access to a specialist for consultation [3, 13, 14].

Telemedicine has been used for several decades to connect lower cadre health workers in remote areas to specialists far away [15, 16]. However, this service requires significant resources to function in a sustainable manner. Mobile health (mHealth) applications are cheaper and may have the same potential to bridge the knowledge and skills gap among FLHW to save lives [17]. Various mHealth applications designed to improve management of sick newborns have been tested and show promise [18–20]. Applications have also been extended to include training of FLHW in retention of knowledge and skills for managing newborns [21], patient follow-up, and communication of critical laboratory results [22, 23], creating a vibrant and innovative landscape in mHealth. Most of these interventions target the patient with few directed towards capacity development of practicing health workers [24–27].

Smart phones are now widely available in resource limited settings and, for the health workers in sub Saharan Africa [28, 29], this presents an opportunity to support mHealth applications. However, there are few innovations on the continent that have been developed to take advantage of these advancements. We hypothesized that a tool to aid FLHW in providing care for sick newborns might perform comparably to a specialist pediatrician. Therefore, we developed and tested an automated text message system called PRISMS (Protecting Infants Remotely by Short Message Service (SMS)). PRISMS is a cellphone-based platform with management algorithms designed to mimic those of a specialist pediatrician. PRISMS uses routine clinical assessment findings to provide newborn care management suggestions to frontline health workers by text message. The purpose of this study was to determine the feasibility, acceptability and efficacy of PRISMS in terms of its performance in diagnosis and management of newborns compared to specialist pediatricians, using simulated newborn scenarios as an initial step to PRISMS validation.

## Methods

### Development and functionality of PRISMS

PRISMS is composed of a remote automated server and a phone application that runs on an Android device. The phone application is comprised of a phone-based-form into which clinical assessment findings are entered. All fields on the form have to be completed for the message “send button” at the bottom of the form to become active. The health worker will not be able to send assessment findings to the server without entering missing information. The clinical assessment findings are entered as raw numerical data for the case of age, gestational age, temperature, respiratory rate, and heart rate. The rest of the parameters are entered as a selection from a dropdown list of predetermined response categories. Once an assessment form is completely filled, and the send button clicked to submit findings, the PRISMS application utilizes native functionalities of the Android device to send a formatted text via SMS to the PRISMS server. At the server, (available 24 h a day) the formatted text was received by a 2-Way SMS Gateway and sent to an algorithm script. Feedback from the algorithm script was processed, prepackaged and sent via the same SMS Gateway to the PRISMS user as proposed clinical management plans. These clinical management plans are based on predetermined server algorithms extensively tested in lab settings by the study team. Our study team included four experienced medical doctors (two Canadian neonatologists, one Ugandan pediatrician and an epidemiologist) and a Ugandan computer programmer. The pediatricians on the study team did not participate in assessing the newborn cases using PRISMS in this study. PRISMS uses an algorithm for clinical assessment adapted from the Canadian Acute Care of at Risk Newborns (ACoRN) Primary Survey [30], and World Health Organization Newborn Guidelines [31].

### Development of newborn case scenarios

A group of four postgraduate trainees in the Masters of Pediatrics program at Mbarara University of Science and Technology (MUST) Department of Pediatrics developed 12 different newborn case scenarios based on clinical cases they had seen on the neonatal unit in Mbarara Regional Referral Hospital (MRRH). MRRH is a tertiary health care facility with a catchment area of approximately 5 million people. The study team checked all cases for completeness. A case was considered complete if it contained at least a short descriptive clinical history, patient age, weight, gestational age, temperature, skin color, heart rate, capillary refill time, degree of dehydration, respiratory rate, presence or absence of chest-in-drawing, presence or absence of noisy breathing, convulsions at the time of clinical examination, breast feeding ability and jaundice assessment (Additional file 1, details all 12 case scenarios). The results for jaundice assessment were provided and classified as absent, mild jaundice or deep jaundice. Presence of jaundice within 24 h of birth and persistence of jaundice after 3 weeks of birth were made as other selectable jaundice characteristics. The ability to breastfeed was categorized as breast feeding well, breastfeeding poorly or unable to breastfeed. PRISMS recommended clinical management suggestions to different assessment-finding-combinations were reviewed for alignment to existing newborn care guidelines by two Canadian neonatologists and one Ugandan Pediatrician.

### Participant recruitment and familiarization to PRISMS

Using convenience sampling, we recruited volunteer pediatricians involved in regular clinical management of newborn babies from four referral hospitals in southwestern, central and eastern Uganda, regardless of the time since their training. We used a convenience sample because of the limited number of pediatricians in the country. We selected our study participants from a pool estimated to be 16 pediatricians at the hospitals we contacted. We used a computer random number generator to assign each pediatrician six of the twelve newborn cases. Each of the twelve cases was equally likely to be selected.

Pediatricians were requested to develop comprehensive clinical case management plans for each of the six randomly selected newborn case scenarios on a case-specific hardcopy clinical management form.

Each pediatrician then received a 10-min orientation and training on how to use the PRISMS platform. We enhanced familiarity with the PRISMS phone application by allowing each pediatrician to input the assessment findings from the other six of the 12 case scenarios that were not randomly selected for pediatrician management into PRISMS to obtain PRISMS suggested clinical management plans. Pediatricians were then asked to use the PRISMS application to obtain clinical management plans for the six cases that they had previously managed without PRISMS.

We categorized PRISMS and pediatrician suggested clinical case managements into four broad classes: 1) thermal care interventions, 2) laboratory investigations, 3) medical treatment, and 4) other management interventions. Data were entered into EpiInfo and analyzed using Stata version 12 (College Station, Texas). We determined agreement between pediatrician and PRISMS suggested clinical management plans using the percentage agreement and the kappa statistic. We used the two approaches to assess agreement because the percentage agreement alone, although easy to interpret, has potential to overestimate agreement to include that due to chance. The kappa statistic is adjusted to measure agreement beyond that expected due to chance and a kappa below 0.4 is considered to be poor [32–34]. The feasibility and acceptability of PRISMS among the users was measured with user experience and satisfaction surveys with a number of items on the Likert scales developed by the research team. The Likert scale scores ranged from 1 to 5 with 1 = very bad and 5 = very good. We used Cronbach’s alpha to measure the internal consistence of these scales and report the scores.

### Human subject issues

All pediatricians enrolled in the study provided written informed consent. No personal identifiers were collected. The study was approved by both Mbarara University of Science and Technology Research Ethics Committee and the Uganda National Council of Science and Technology.

## Results

Seven pediatricians, two males and five females, conducted a total of 42 newborn case scenario assessments and made managements plans for them. All pediatricians received their pediatric training in Uganda and had a mean pediatrics clinical care experience of 5.9 years (95% CI: 2.63 – 9.08). All pediatricians (7/7) had been exposed to Helping Babies Breathe (HBB) and Essential Care for Every Baby (ECEB) [35] as trainees and trainers.

### Case scenario characteristics

The 42 cases (Table 1) had different combinations of clinical signs and symptoms. Fever (axillary temperature > 37.5 °C) and hypothermia (temperature < 36.5) was present in 35.7% (15/42) and 45.2% (19/42) of cases respectively. Fast breathing (respiratory rate greater than 60 breathes per minute) was present among 52.3% of all case scenarios. Half of cases with jaundice had deep jaundice and the rest of jaundiced cases had mild jaundice. Although we had 12 independent cases, repeated assessments were done. In the results, we present in Table 1, the details of frequency of occurrence of different clinical signs among the 42 case scenario assessments selected from the pool of 12 cases managed by the 7 pediatricians.

**Table 1:** Table showing the frequency of clinical signs among 42 case scenarios managed by pediatricians and PRISMS phone application

Clinical sign or symptom	Frequency of occurrence % (n/N)
Low birth weight (weight less than 2500 g)	31% (13/42)
Fever (temperature greater than 37.5 °C)	35.7% (15/42)
Hypothermia (temperature less than 36.5 °C	45.2% (19/42)
Severe hypothermia (Temperature less than 35.5 °C)	21.4% (9/42)
Convulsions present at presentation	9.5% (4/42)
Fast breathing (Rate greater than 60 per minute)	52.3% (22/42)
Chest in-drawing	23.8% (10/42)
Noisy breathing	4.8% (2/42)
Poor breastfeeding	26.2% (11/42)
Jaundice	19% (8/42)

### User experience

Overall, PRISMS was rated as feasible based on the user experience and satisfaction. The overall mean score for user experience (Table 2) was 4.1 out of a potential maximum of 5 indicating an overall good experience. The scores on the individual items ranged between 3.8 for the item on time to complete filling information into PRISMS application form and 4.3 for ease of use of PRISMS.

**Table 2:** Table showing summary scores (range 1 = very bad, 5 = very good) for items on the user experience scale for using PRISMS among Pediatricians (*n* = 7)

Parameter of user experience	Mean	SD^a^
Time to receive management suggestion	4	1.2
Time to complete filling information into phone data form	3.8	0.4
Length of phone data form	4.3	0.5
Completeness of management information provided	4	0.8
Ease of use of PRISMS application	4.3	0.8

^a^*SD standard deviation. Overall mean score for this scale = 4.1 The Cronbach’s alpha for this scale was 0.80*

### Pediatrician satisfaction with PRISMS

We assessed satisfaction using 8 items as shown in Table 3. The item with the maximum score was “Investigations provided by PRISMS were adequate” with a score of 4.1 out of a maximum score of 5. The lowest score was 3.4 for the item “PRISMS provides comprehensive newborn management”. The overall mean score was 3.8 out of a maximum score of 5.

**Table 3:** Table showing summary scores (range 1 = strongly disagree, 5 = strongly agree) for user satisfaction scale using PRISMS among Pediatricians (*n* = 7)

Parameter	Mean	SD^a^
Prisms provides sufficient management of the newborn	3.7	1.1
I will use PRISMS in the care of babies	4	0.8
There were aspects of care I missed that I got reminded by PRISMS	4	1.0
PRISMS provides comprehensive newborn management	3.4	0.8
The investigations provided by PRISMS were adequate	4.1	0.4
PRISMS should be used by all Health workers	3.7	1.0
PRISMS can be used in Hospitals	4.1	0.7
The cases were easy to manage	3.6	0.8

*Overall mean score for this scale = 3.8 (SD = 0.6) The Cronbach’s alpha for this scale was 0.83.*

^a^*SD standard deviation*

When asked whether “PRISMS can only be used outside hospitals”, the mean Likert score for this question was 2.3 (SD = 1.1). Respondents’ disagreement with restricting use suggests support for use across a variety of health facility settings.

### Clinical management agreement is seen in Table 4

Statistically significant concordance in pediatrician and PRISMS for clinical management was obtained for prolonged skin to skin care, intravenous (IV) 10% dextrose administration, blood transfusion, phototherapy, exchange transfusion, and investigations for jaundice. However, there was lack of agreement with certain components of management namely: decision to reduce clothing, doing a complete blood count, blood culture, lumbar puncture and use of antibiotics.

**Table 4:** Table showing level of agreement in newborn case management between PRISMS and Pediatricians on 42 case assessments

Comparison of thermal care interventions between pediatrician and PRISMS				Comparison of investigation recommendations between pediatrician and PRISMS			
Intervention	Agreement (%)	Kappa	*p*-value	Recommendation	Agreement (%)	Kappa	p-Value
Remove wet cloths^b^	54.8	0.00	0.5000	Complete blood count	50	0.04	0.3036
Prolonged skin-skin- care (KMC)	66.7	0.29	0.0092	Blood culture	45.2	0.00	0.5000
Cover with blankets and hat	64.3	0.23	0.0104	Random blood sugar	47.6	0.14	0.0682
Reduce clothing^b^	–	TD	–	Lumber puncture	31.0	0.07	0.1184
Recheck Temp in 1 Hour^b^	–	TD	–	Coombs test	95.2	0.64	0.0000
				Bilirubin total and differential	97.6	0.84	0.0000
**Comparison of treatment recommendations between pediatrician and PRISMS**				**Comparison of management Interventions between pediatrician and PRISMS**			
Intervention	Agreement (%)	Kappa	p-Value	Intervention	Agreement (%)	Kappa	p-Value
IV 10% Dextrose bolus	73.8	0.50	0.0001	Check / position airway	57.1	0.01	0.5
IV Normal Saline	73.8	0.45	0.0014	Bag-valve-mask ventilation	90.5	−0.05	0.6270
Antibiotics^a^	73.8	0.11	0.2410	Alternative feeding (NGT/EBM)	64.3	0.34	0.0036
Anticonvulsants	100	1.0	0.0000	Supplemental oxygen	69.1	0.40	0.0034
Blood Transfusion	81.0	0.60	0.0000				
Phototherapy	90.5	0.62	0.0000				
Exchange Transfusion	–	TA	–				

*TD* Total (100%) Disagreement. *TA* Total (100%) Agreement

^a^Pediatricians were less likely to prescribe antibiotics compared to PRISMS

^b^Pediatricians were less likely to remove wet clothes, reduce clothing and recheck temperature

## Discussion

We designed and tested a novel cell phone platform (PRISMS) to assist health workers with no specialty training in neonatal care to manage sick newborns in a resource limited setting. Our results also show there was a good level of agreement in the management plans proposed by PRISMS and the pediatrician, and there were areas where the pediatrician felt PRISMS enhanced their prior clinical management plans.

For many countries in resource limited settings, majority of patients seek health care at lower level health facilities. In these facilities they often receive care from non-specialized FLHWs [36]. Our next step will be to investigate use of PRISMS in these frontline health workers with an aim to strengthen their ability to provide newborn care. We chose to start with a higher level of specialty in order to test the performance of the tool against these specialists as our stated gold standard to examine its validity.

We assessed PRISMS to ensure its functionality to established standards of care. This care standards included validated newborn danger signs predictive of severe illness as detailed by the Young Infants Clinical Signs Study Group [37]. We noted that for interventions related to thermal care, PRISMS and the pediatricians were more likely to disagree compared to other components of management. For two aspects of thermal care management (reducing clothing and rechecking temperature after one hour), there was total disagreement between PRISMS and Pediatrician. All case scenarios with fever (15/42) had no pediatrician recommendation for reduction of clothing while PRISMS recommended clothing reduction for all. None of the pediatricians recommended a recheck of temperature one hour following any thermal intervention provided to febrile or hypothermic cases. These thermal care management disagreements were reported by pediatricians as management omissions when they compared their suggested care to that of PRISMS. The management of febrile babies with exposure/ reduction of clothing, and of hypothermic babies with removal of any wet clothing, covering with dry warm clothing and use of skin-to-skin contact followed by a repeat temperature measurement in one hour is a recommended thermal care measure [35]. PRISMS was more adherent to these thermal recommendations than the Pediatricians.

We observed management options where pediatricians had complete agreement with PRISMS. The item with complete agreement was exchange transfusion although it should be noted that this is a relatively uncommon aspect of clinical care which will not be able to be carried out without patient transfer when PRISMS is next tested in smaller health centers. The complete agreement could be explained by the fact that we enrolled pediatricians from tertiary referral centers where exchange transfusion is commonly offered as a specialist’s procedure. The pediatricians are expected to be familiar with the procedure. There were pediatricians that recommended investigations such as c-reactive protein (CRP) measurement for babies with suspected infections that PRISMS was not recommending. Though CRP may indicate likelihood for sepsis, PRISMS did not recommend its use for patients with danger signs. The developers of the algorithm felt CRP was not critical to recommend as majority of newborn care facilities in developing countries often do not have facilities to test for CRP. Pediatricians were less likely to recommend antibiotics compared to PRISMS. This was because PRISMS would recommend antibiotics to all babies with any clinical signs predictive of severe illness [37]. Some Pediatricians on the other hand were cautious to recommend antibiotics before investigation results, such as for CRP when signs predictive of severe illness were present. These differences in approach contributed to the level of agreement observed between PRISMS and Pediatricians for administration of antibiotics.

Mobile applications have been used to improve skilled attendance at delivery [25], and follow up infants for other outcomes such as breastfeeding and perinatal mortality [24, 38]. Existing interventions have targeted the patients, but very few have targeted the health worker [24–27]. Health worker targeted electronic interventions have mainly been for management of childhood illnesses with limited focus on newborn care [39–41]. A strength of our study is that our mobile application is built on the android platform allowing wide scale deployment due to increasing android device availability.

Our study sets the pace for quality of care improvement and standardization of newborn care assessment and care planning. Such care benefits have been realized with the use of electronic systems for Integrated Management of Childhood Illnesses and Community Case Management of Malaria, Pneumonia and Diarrhea [39, 41]. These have demonstrated better adherence to protocol, and improved clinical care outcomes for infants and under-five children both at facility and community levels compared to paper based versions [40, 42].

The time taken to receive clinical management plans after completing the PRISMS assessment form had an average satisfaction score of 4. There were times when text messages from the server delayed to be received by PRISMS users due to telephone network challenges. We have already implemented an inbuilt server algorithm that guarantees provision of clinical management plans in less than 8 s independent of internet and telephone networks. Therefore, PRISMS use in health facilities for the generation of clinical management plans no longer requires internet or telephone network connectivity. However, for remote synchronization of data from PRISMS devices to the backend server, internet connectivity is required.

With the 4.1 average score on the item “PRISMS can be used in hospitals”, this seems like PRISMS will be a likely successful addition to clinical care in these settings. Hospitals are associated with greater investigative capacity that are seldom available in lower unit health facilities. We have restructured the clinical management suggestions provided by PRISMS to be applicable in higher level facilities with more investigative capacity. For example, we would state “consider full blood count, blood culture and lumber puncture” for all babies with danger signs. We plan to elect clinical investigation suggestions that are preceded with the word “consider” to refer to management suggestions that are desired if the health facility in which the baby is managed has the ability to provide such investigations.

### Limitations

Our study has limitations. We have tested this application among pediatricians and not among the non-pediatrician frontline health workers such as midwives, nurses, clinical officers and medical officers who provide the greatest bulk of newborn care decisions in Sub-Saharan Africa especially at the lower level health facilities. The lower level facility staff are the ones more likely to need assistance in management of sick newborns.

We have demonstrated feasibility but we now need to test this application using a randomized controlled design among the likely end users to determine its effect on quality of newborn care and newborn care outcomes. A randomized cluster trial for this inquiry is ongoing.

This application assumes that the health worker has adequate clinical skills to identify key clinical signs and symptoms upon which the clinical management algorithm is based. We are aware of some limitations in clinical skills among lower level cadres and even pediatricians due to knowledge and skills decay. One way to overcome this is to provide refresher training in clinical assessment prior to implementation of the intervention.

These finding are based on case assessments sampled from twelve different case scenarios and these may not be representative of the entire breadth of different newborn cases. In addition, recommendations for clinical care change with time and the algorithm will need to be kept up to date.

## Conclusion

We have successfully developed, tested and demonstrated feasibility and acceptability of a mobile platform to manage sick newborns. This application has demonstrated a reminder function and acceptable level of agreement with pediatrician suggested clinical case managements. We acknowledge that the level of agreement was low in some aspects of management.

We plan to test the acceptability and utilization of this application on a larger scale with more frontline healthcare workers. On this large scale, we also propose to assess the impact of this intervention on clinical endpoints such as neonatal mortality.

## Supplementary information

**Additional file 1.** List of case scenarios used in the comparative study of clinical case managements between 7 pediatricians and PRISMS.

## Data Availability

The datasets generated and/or analyzed during the current study are not publicly available due to ongoing processes to complete securing intellectual property for the PRISMS technology but are available from the corresponding author on reasonable request.

## Abbreviations

ACoRN: Acute Care of at-Risk Newborns
ECEB: Essential Care for Every Baby
FLHW: Front Line Health Worker
HBB: Helping Babies Breathe
IV: Intravenous
mHealth: Mobile Health
MRRH: Mbarara Regional Referral Hospital
MUST: Mbarara University of Science and Technology
PRISMS: Protecting Infants Remotely by Short Message Service
SMS: Short Message Service
