# Supplementary material for: First-step validation of a text message-based application for newborn clinical management among pediatricians
Source: BMC Pediatr. 2020 Aug 27;20:406. doi: 10.1186/s12887-020-02307-2 (PMC7450570; doi:10.1186/s12887-020-02307-2)
Supplement: Supplementary file 1 — Additional file 1. List of case scenarios used in the comparative study of clinical case managements between 7 pediatricians and PRISMS. [file 12887_2020_2307_MOESM1_ESM.docx]

**Additional file 1:** List of case scenarios used in the comparative study of clinical case managements between 7 pediatricians and PRISMS.

**Case 001**

| A 5days old baby was born by spontaneous vaginal delivery at Mbarara Regional Referral Hospital at 38 weeks of gestation. The Apgar score was 9 at one minute and 10 at 5 minutes. Two days ago, the baby developed a high-grade fever associated with poor feeding. No excessive crying, no convulsions and has no difficulty in breathing. | | | |
| --- | --- | --- | --- |
| On examination: |  |  |  |
| Baby had yellowing of skin and sclera, pallor of conjunctiva and soles of the feet | | | |
| Weight - | 2.7kg | Temperature | 36.8 ^0^C |
| Heart rate - | 137 b/min | Color - | Pale looking |
| Capillary refill time - | 2 seconds | Degree of dehydration - | Some dehydration |
| Respiratory rate - | 56 b/min | Chest in-drawing - | Absent |
| Convulsions - | Absent | Noisy breathing - | Absent |
| Ability to feed - | Breast feeding well | Jaundice | Deeply jaundiced |
| The chest is clear on auscultation and heart sounds are heard and considered normal. There were no other abnormal physical findings on examination. | | | |

**Cases 002**

| A three-hour old baby was delivered by spontaneous vaginal delivery at 40 weeks of gestation after 12 hours of labor. The Apgar score at 1 minute was 4/10 and at 5 minutes was 8/10. Baby has been unable to breastfeed since birth, and reported to have had a high temperature. 30 minutes ago baby had twitching of the left leg associated with repeated side-ward movement of the eyes that lasted about 7 minutes. | | | |
| --- | --- | --- | --- |
| On examination: |  |  |  |
| Baby looks term, alert and actively moving both upper and lower limbs | | | |
| Weight - | 3.4 kg | Temperature - | 38.7 ^0^C |
| Heart rate - | 157 b/min | Color - | Pink looking |
| Capillary refill time - | 2 seconds | Degree of dehydration - | Well hydrated |
| Respiratory rate - | 49 b/min | Chest in-drawing - | Absent |
| Convulsions - | Not convulsing now | Noisy breathing - | Absent |
| Ability to feed - | Breast feeds poorly | Jaundice - | Absent |
| Chest had normal breath sounds and heart sound one and two were heard and found normal | | | |

**Case 003**

| A mother delivered a term baby 3 days ago at 39 weeks of gestation, the baby has been well for the last three days. However, the mother reports that something seems not to be right with the baby but mother is not sure what. Baby seems to be breastfeeding well but mother is not sure since this is the mother’s first baby. | | | |
| --- | --- | --- | --- |
| On examination: |  |  |  |
| Well looking baby resting calmly on the mothers laps with no respiratory distress | | | |
| Weight - | 1.8 kg | Temperature | 35.7 ^0^C |
| Heart rate - | 145 b/min | Color - | Pink |
| Capillary refill time - | 3 seconds | Degree of dehydration - | Well hydrated |
| Respiratory rate - | 47 b/min | Chest in-drawing - | Absent |
| Convulsions - | Not convulsing | Noisy breathing - | Absent |
| Ability to feed - | Breast feeds well | Jaundice - | Absent |
| Mother looks anxious | | | |

**Case 004**

| A one-day old baby was born at 32 weeks of gestation, after 7 hours of maternal mild lower abdominal pain. Mother had fever during the labor but no fowl smelling vaginal discharge. Baby cried soon after birth. Baby has been breastfeeding but poorly, however, baby has been unable to feed for the last 3 hours. | | | |
| --- | --- | --- | --- |
| On examination: |  |  |  |
| Small looking baby breathing calmly with minimal limp movement | | | |
| Weight - | 1.7 kg | Temperature | 35.2 ^0^C |
| Heart rate - | 102 b/min | Color - | Pink |
| Capillary refill time - | 2 seconds | Degree of dehydration - | Some dehydration |
| Respiratory rate - | 60 b/ min | Chest in-drawing - | Absent |
| Convulsions - | Absent | Noisy breathing - | Absent |
| Ability to feed - | Unable to suckle | Jaundice - | Absent |
|  | | | |

**Case 005**

| A Baby was delivered by spontaneous vaginal delivery at 39 weeks of amenorrhea a Regional Referral Hospital 3 days ago. Mother reports baby had been well and received some drops in the mouth and an injection on the right shoulder at the immunization clinic. Baby started bleeding from the cord 20 minutes ago. A bandage was placed at the cord in the nearby clinic but baby seem to be weaker now. | | | |
| --- | --- | --- | --- |
| On examination: |  |  |  |
| Weak looking pale baby wrapped in blood stained towels | | | |
| Weight - | 4 kg | Temperature | 36 ^0^C |
| Heart rate - | 180 b/min | Color - | Pale |
| Capillary refill time - | 4 seconds | Degree of dehydration - | Some dehydration |
| Respiratory rate - | 43 b/min | Chest in-drawing - | Present |
| Convulsions - | Absent | Noisy breathing - | Absent |
| Ability to feed - | Unable to breastfeed | Jaundice - | Absent |
| The anterior fontanel is tense and bulging and the neck is soft | | | |

**Case 006**

| A three-week-old baby was brought by the mother after 2 days of high temperature and poor breastfeeding. Baby was treated in a clinic for cough and fever with syrup amoxicillin without any improvement. This morning baby had convulsions involving a finger of the left hand and one side of the mouth. Baby has been feeding poorly. Baby was delivered at home by the grandmother and has otherwise been well. Labor and delivery was uneventful and baby was delivered at 38 weeks of gestation. | | | |
| --- | --- | --- | --- |
| On examination: |  |  |  |
| Pale and sick looking baby with continuous twitching of the finger and corner of the mouth. | | | |
| Weight - | 3.9 kg | Temperature | 37.3 ^0^C |
| Heart rate - | 127 b/min | Color - | Pale |
| Capillary refill time - | 3 seconds | Degree of dehydration - | Mild |
| Respiratory rate - | 82 b/min | Chest in-drawing - | Absent |
| Convulsions - | Present | Noisy breathing - | Absent |
| Ability to feed - | Poor | Jaundice - | Absent |
| Examination of the lungs and heart sounds revealed normal findings | | | |

**Case 007**

| A 5-day old baby was born to a para 2 mother at 38 weeks of pregnancy, the pregnancy was uneventful (no problems). Labor took 14 hours and baby was delivered by breach extraction at Mbarara Regional Referral Hospital. Apgar score was 5 at one minute and 8 at 5 minutes. Baby required resuscitation by bag and mask ventilation for about 20 minutes. | | | |
| --- | --- | --- | --- |
| On examination: |  |  |  |
| Normal sized baby in respiratory distress | | | |
| Weight - | 3.4 kg | Temperature | 36.1 ^0^C |
| Heart rate - | 159 b/min | Color - | Pink |
| Capillary refill time - | 3 seconds | Degree of dehydration - | None |
| Respiratory rate - | 82 b/min | Chest in-drawing - | Present |
| Convulsions - | Absent | Noisy breathing - | Present |
| Ability to feed - | Poor | Jaundice | Absent |
|  | | | |

**Case 008**

| A 14-day old baby was born to a mother as a second born at 30 weeks of pregnancy due to early rupture of membranes. Mother had no fevers during the labor or in the days before onset of labor. Baby’s APGAR score was 6 at 1 minute and 9 at 5 minutes. Baby was admitted in the ward and fed by Nasogastric tube for one week before discharge on both breastfeeding and supplemental Nasogastric tube feeding. Now baby presents with a 2-day history of abdominal distension, vomiting of greenish-yellow substance and failure to breastfeed. | | | |
| --- | --- | --- | --- |
| On examination: |  |  |  |
| Sick looking baby with distended, tense and shiny abdomen and shallow rapid breaths | | | |
| Weight - | 1.6 kg | Temperature - | 37.9 ^0^C |
| Heart rate - | 158 b/min | Color - | Pale |
| Capillary refill time - | 4 seconds | Degree of dehydration - | Some |
| Respiratory rate - | 77 b/min | Chest in-drawing - | Absent |
| Convulsions - | Absent | Noisy breathing - | Absent |
| Ability to feed - | Unable | Jaundice - | Mild |
| Bowels sounds were decreased and dippers had a little blood-stained stool | | | |

**Case 009**

| A six-day old neonate was delivered vaginally at 40 weeks of gestation from a peripheral health center with no complications. Baby was taken for false tooth extraction a day prior to admission, it resulted into significant bleeding from the gums associated with passing melena stools. Later, baby developed weakness and inability to breast feed and is less active today. | | | |
| --- | --- | --- | --- |
| On examination: |  |  |  |
| Very weak looking baby | | | |
| Weight - | 3.8 kg | Temperature | 38.7 ^0^C |
| Heart rate - | 180 b/min | Color - | Very pale |
| Capillary refill time - | 4 seconds | Degree of dehydration - | Some dehydration |
| Respiratory rate - | 88 b/min | Chest in-drawing - | Absent |
| Convulsions - | Absent | Noisy breathing - | Absent |
| Ability to feed - | Unable to breast feed | Jaundice - | Absent |
| The liver is palpable below the costal margin | | | |

**Case 010**

| A one-week old neonate born at 37 weeks of gestation with no complications, and was initiated to breast feed. Baby developed a mild papular rash on the face that was treated from home by application of local herbs. Two days to admission, the baby developed a generalized rash with peeling of the whole skin, a high-grade fever and poor breast feeding. | | | |
| --- | --- | --- | --- |
| On examination: |  |  |  |
| Sick looking baby, has good spontaneous movements of both upper and lower limbs | | | |
| Weight - | 2.8 kg | Temperature | 39 ^0^C |
| Heart rate - | 167 b/min | Color - | Pink |
| Capillary refill time - | 3 seconds | Degree of dehydration - | None |
| Respiratory rate - | 68 b/min | Chest in-drawing - | Absent |
| Convulsions - | Absent | Noisy breathing - | Absent |
| Ability to feed - | Well | Jaundice - | Absent |
| Has a generalized pustular rush with skin peeling and areas of mild skin bleeding | | | |

**Case 011**

| A two-day old preterm baby was born at 31 weeks of gestation by caesarian section due to preterm, prolonged rupture of membranes. Baby cried at birth but was cyanosed and got difficulty in breathing a few minutes after birth. Both the cyanosis and difficulty in breathing didn't improve. | | | |
| --- | --- | --- | --- |
| On examination: |  |  |  |
| Small sick looking baby with central and peripheral cyanosis. | | | |
| Weight - | 1.3 kg | Temperature | 35.2 ^0^C |
| Heart rate - | 178 b/min | Color - | Bluish |
| Capillary refill time - | 2 seconds | Degree of dehydration - | None |
| Respiratory rate - | 82 b/min | Chest in-drawing - | Present |
| Convulsions - | Absent | Noisy breathing - | Absent |
| Ability to feed - | Poor | Jaundice | Absent |
| Lung fields had bilateral fine crepitations. Heart sounds are heard with a systolic murmur at the upper left border of the sternum. | | | |

**Case 012**

| A 30 minutes old term neonate was delivered by assisted vaginal delivery at 42 weeks of gestation due to prolonged labor. Mother had been referred from a peripheral health center after laboring for 2 days without good progress. Baby was born floppy, cyanosed, didn't cry and failed to breathe. Was resuscitated by bag and mask and baby started gasping. | | | |
| --- | --- | --- | --- |
| On examination: |  |  |  |
| Floppy unconscious term baby with caput, peripheral cyanosis and a cold clammy skin | | | |
| Weight - | 3.9 kg | Temperature | 33.2 ^0^C |
| Heart rate - | 70 b/min | Color - | Pale dusky skin |
| Capillary refill time - | 3 seconds | Degree of dehydration - | None |
| Respiratory rate - | ~ 12 b/min - gasping | Chest in-drawing - | Absent |
| Convulsions - | Absent | Noisy breathing - | Absent |
| Ability to feed - | Unable | Jaundice | Absent |
| Baby has irregular gasping respirations | | | |
